# Barriers to blockchain adoption for supply chain finance: the case of Indian SMEs

**DOI:** 10.1007/s10660-022-09566-4

**Published:** 2022-07-20

**Authors:** Jaspreet Kaur, Satish Kumar, Balkrishna E. Narkhede, Marina Dabić, Ajay Pal Singh Rathore, Rohit Joshi

**Affiliations:** 1 Department of Management Studies, Malaviya National Institute of Technology, Jaipur, Rajasthan 302017 India; 2 Faculty of Business, Swinburne University of Technology Sarawak, Kuching, Malaysia; 3 Department of Operations and Supply Chain Management, National Institute of Industrial Engineering (NITIE), Vihar Lake, Powai, Mumbai, Maharashtra 400087 India; 4 Faculty of Economics and Business, University of Zagreb, Zagreb, Croatia; 5 Nottingham Trent University, Nottingham, UK; 6 Department of Mechanical Engineering, Malaviya National Institute of Technology, Jaipur, Rajasthan India; 7 Operations and Quantitative Techniques Area, Indian Institute of Management Shillong, Mayurbhanj Complex, Nongthymmai, Shillong, 793 014 India

**Keywords:** Blockchain technology, Supply chain finance, SME, Barriers, Fuzzy-AHP, Fuzzy-DEMATEL, Sensitivity analysis

## Abstract

Small and medium enterprises (SMEs) in India are suffering from the long-standing challenges related to asymmetric information, high transaction costs, SMEs’ opacity and limited access to credit. Blockchain technology, which is still in its infancy in terms of adoption in India, can facilitate SMEs to counter these challenges. Fuelled by this motivation, the study aims to investigate the significant barriers to blockchain adoption in supply chain finance practices by Indian SMEs. Using fuzzy-analytic hierarchy process, sensitivity analysis, and fuzzy-decision-making trial and evaluation laboratory this paper identifies the blockchain barriers, prioritises them and examine their cause and effect relationships. The results of the study indicate that technology barriers are the most influential barriers that impede blockchain adoption. The findings will help the policymakers and practitioners to take suitable measures to overcome these barriers and fuel the adoption of blockchain in Indian SMEs.

## Introduction

**Fig. 1:**
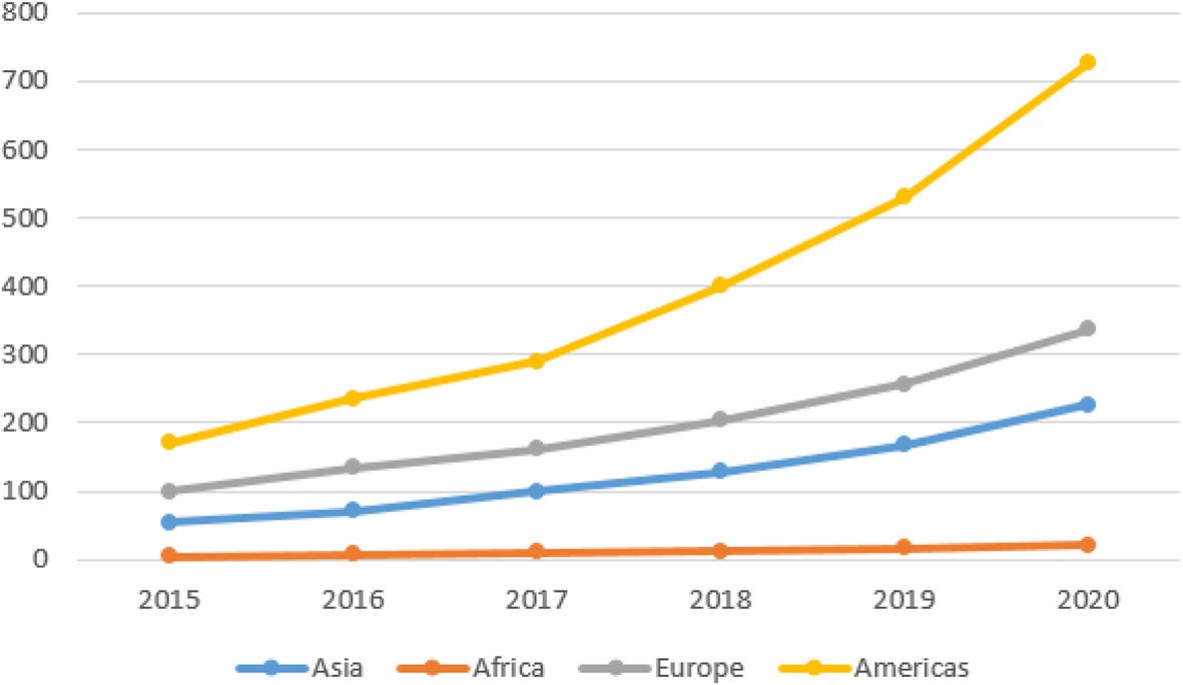
Global SCF volumes 2015–2020 (*Source*: BCR, 2021 [8])

Supply chain finance (SCF) is an approach for two or more supply chain partners and an external service provider to create value by planning, steering, and controlling the flow of financial resources on an inter-organizational level [1]. It is located at the intersection of supply chain management, logistics and finance. SCF emerged in the literature of supply chain management and gained further interest and recognition from researchers after the financial turmoil caused by the global financial crisis of 2008 [2]. Moreover, after COVID-19 hit the global economy, firms started turning towards SCF solutions to stabilise their net working capital and maintain solvency. The global SCF volumes have shown a significant growth rate in 2020 over 2019 [3]. In this context, Fig. 1 illustrates the increasing global SCF volumes in the last six years. Supply chain finance plays a pivotal role in expanding the scope of financing for SMEs and reducing their cost of capital [4, 5]. Although SCF is an efficient method for lowering the financing cost, supply chain finance solutions are highly manual and siloed, inducing high overhead cost and lack of visibility [6]. Automation of processes in financial supply chains is imperative for developing the SCF market because SCF solutions rely on effective and fast processing of the supply chain data [7].

Blockchain technology can bring new levels of collaboration among the supply chain actors and accelerate the cash flows through the supply chain [9]. It is a decentralised, shared and cryptographically unaltered ledger that can record and maintain the history of digital transactions [10–12]. The blockchain-based system does not need intermediaries, which reduces the transaction cost and eradicates human-induced errors, risks, and loss of time [13].

Traditional SCF processes suffer from inefficiencies in the financial settlements in the supply chain. Although SMEs have digitised their processes to some extent, the processing of financial transactions remains in traditional paper form in most organisations, leading to delay in payment, greater days of sales outstanding, and ultimately raising the demand for working capital [14]. Blockchain technology can overcome these challenges by providing a tamper-proof history of transactions leading to increased transparency in financial supply chains [15]. Furthermore, there may be distortion or falsification of documents, information, or cash in traditional SCF practices. However, blockchain assures fairness and allows for secure authentication of the transactions. It has great potential to build trust and boost supply chains’ financing ability, which is conducive to promoting financial development in SMEs in India [14].

The commercial application projects in this area are already gaining traction. The technology giant IBM has teamed up with one of the largest logistics service providers, Maersk Line, to create a blockchain-based solution to digitise the global, cross-border supply chains. China-based fintech firms Dianrong and FnConn have launched the blockchain-based SCF platform to secure funding for SMEs in China [16]. Moreover, several start-ups have started working in the area of blockchain-based bills of lading, letters of credit, factoring, and reverse factoring [17].

Blockchain technology has good potential to transform the SCF practices in SMEs, but its adoption is still in its infancy [18]. Although the use cases of blockchain-based SCF platforms have increased over the past few years, blockchain technology faces various barriers in adoption by SMEs. India, similar to developed nations, looks forward to unleashing the true potential of blockchain technology to overcome the challenges faced by SMEs. Therefore, an investigation to discover the current barriers faced by Indian SMEs in blockchain adoption in SCF processes is required. The barriers to blockchain adoption in supply chain management are well addressed in the literature [19–23], but none of the studies have focussed on the SCF related barriers in blockchain adoption. To fill the research gap, this study explores the following research objectives (RO): RO1: To identify the blockchain adoption barriers in SCF in SMEs in India; RO2: To prioritise the identified barriers; RO3: To evaluate the consistency in the ranking of the identified barriers; RO4: To identify the interrelationships amongst the identified barriers.

Identifying the adoption barriers would help understand the steps for the successful adoption of blockchain in SCF processes by SMEs. Such information will be helpful to the policy-making bodies, supply chain partners, and government to prepare an appropriate strategy for adopting blockchain in SCF by SMEs. The following section presents a review of literature explaining the relevance of blockchain technology in SCF processes. The research methodology is explained in Sect. 3. Section 4 highlights the data analysis by applying the proposed methodology. The results are portrayed in Sect. 5, while the implications for managers and policymakers are presented in Sect. 6. Finally, Sect. 7 concludes the paper.

## Literature review

### How does blockchain work?

Blockchain is a decentralised, public database that is shared across a network of computers [24]. This network makes constant checks to ensure that all the copies of the database are the same. The records of the transactions are bundled together into blocks and added to the chain [25]. Blockchain is based on the principles of decentralisation, cryptography, and consensus mechanism, which ensure trust in transactions. Blockchain works on a peer-to-peer network in which the transactions can occur without a central server. The cryptographic algorithms used in blockchain ensure confidentiality, integrity, non-repudiation and data authentication [26].

**Fig. 2:**
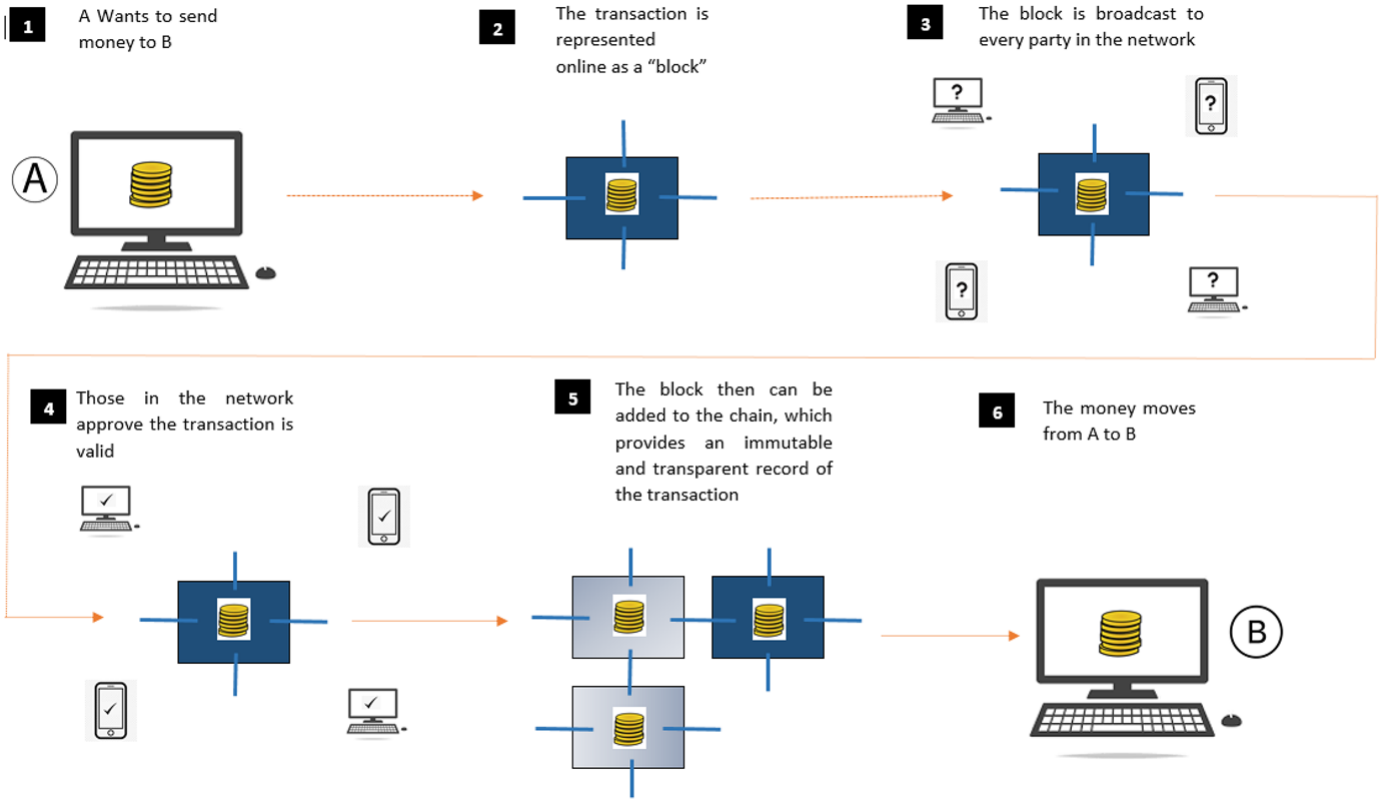
How blockchain transaction works (*Source*: Charfeddine & Umlai [27])

Maintaining trust among a large number of anonymous members on the blockchain is a challenging task due to its open and decentralised architecture. This is where the consensus algorithm comes into play. A consensus algorithm is a mechanism through which all the members of a blockchain network validate the transactions and ensure its accuracy. In this way, blockchain participants can trust the unknown peers in a distributed network and maintain the integrity and security of the data. When new intended transactions are validated and accepted by the network, it is added to a block. A cryptographic algorithm called hashing provides a unique hash value to each block [17]. These blocks also store the previous block’s hash values, which ultimately connect the blocks in a specific order. Figure 2 depicts the steps in a blockchain transaction.

### Supply chain finance: decentralised versus centralised systems

Successful implementation of SCF programs depends on improving software and technology solutions. The automated processes can speed up the cash flows throughout the supply chain. The advent of enterprise resource planning (ERP) systems and the rise of e-invoicing brings a certain level of automation and dematerialisation to B2B processes, enabling faster and more efficient SCF solutions [28].

**Fig. 3:**
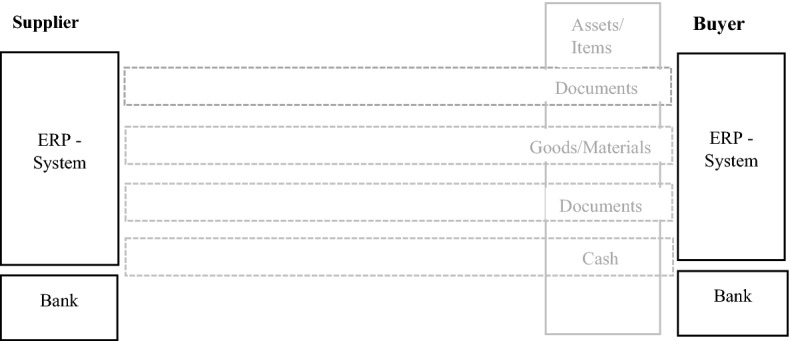
Supply chain layers between buyer and supplier (*Source*: Hofmann et al. [17])

At present, most supply chain operations rely heavily on centralised and stand-alone information management systems, such as ERP systems [20]. These systems have their own drawbacks. The ERP of each organisation is built upon a single database. When two or more organisations enter a transaction, the assets are transferred from the individual database of one organisation to another [29]. The buyers and suppliers deal with each other through four layers of interaction: order processing, shipping, billing & invoicing, and payment [28]. Figure 3 shows the four supply chain layers between a buyer and supplier.

Although ERP systems partially integrate different layers of supply chain processes into one wide application system, isolated operating units can still exist within the organisation [29]. This leads to manual updates from one system to another, requiring reconciliation efforts and increasing human errors.

**Fig. 4:**
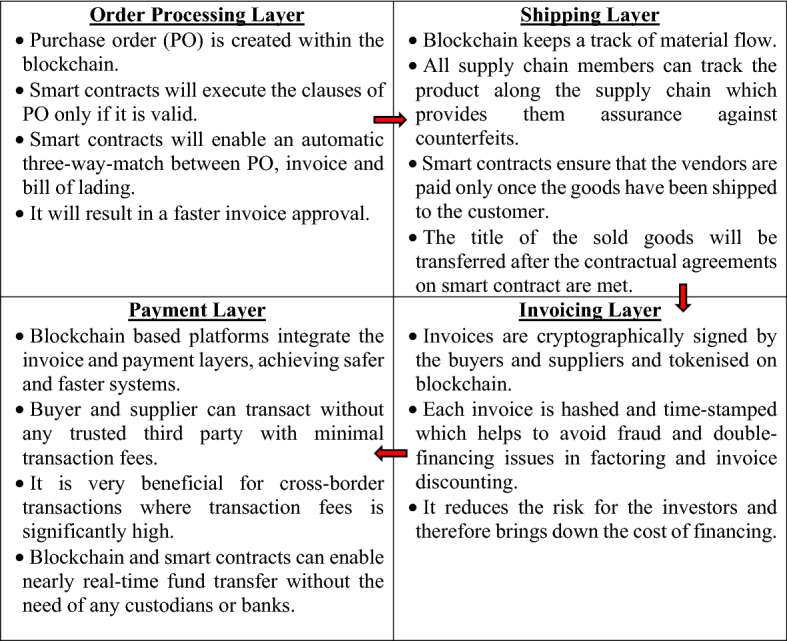
Blockchain-based purchase-to-pay process

On the other hand, if the organisations use blockchain as the underlying technology, information can be recorded and broadcast across all the participants while maintaining a single source of truth [17]. Each participant in the blockchain network will access this information without depending on any centralised system [5]. Figure 4 shows the blockchain-based purchase-to-pay process depicting how blockchain technology transforms all four layers of supply chain processes.

Blockchain enhances the automation of SCF processes by bringing fully digital and signed documents which leads to faster invoice approval [21, 30]. Early approval of the invoice will give a longer time interval for financing it. The financing parties in post-shipment SCF programs, such as factoring, invoice discounting, and reverse factoring, face the risk of double financing and the legal validity of the invoice [17]. Blockchain-driven solutions can mitigate these risks with the help of a digital signature on each e-invoice which creates a unique invoice identifier [30]. Zurich-based start-up ‘Gatechain’ offers blockchain-based factoring and reverse factoring solutions for both domestic and international trade transactions [31]. The compliance check is performed by string comparison in digital documents based on a smart contract in these solutions. It improves efficiency by bringing down costs [8]

The supply chain partners face two key risks in pre-shipment SCF solutions, such as inventory finance and purchase order finance. First, the performance risk of the supplier in fulfilling the purchase order and second, the credit risk of the buyer [17]. Combination and blockchain and IoT solutions can track the physical supply chains to regulate the risk at each stage of the shipping process [30]. Blockchain provides immutable and real-time data available to all the supply chain partners. ‘Skuchain’ is a US-based startup that runs a blockchain-based inventory control and finance program which enhances buyers’ visibility into their inventory and enables the suppliers to acquire the capital at a cheaper cost [31].

### Theoretical foundation

**Fig. 5:**
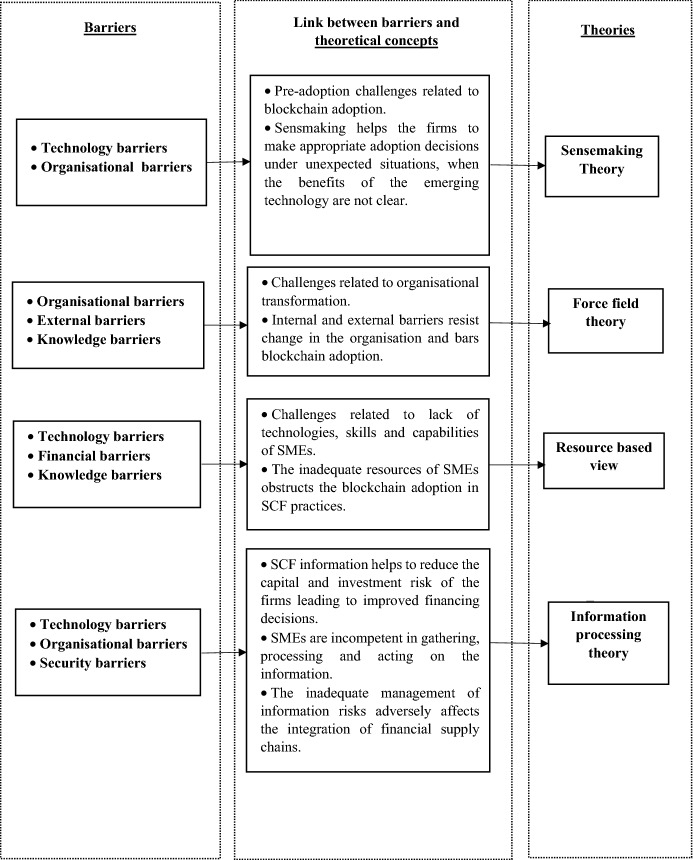
Theoretical framework of the study

In order to develop a comprehensive understanding of blockchain adoption barriers, it is imperative to develop the theoretical foundation of the barriers discussed in the study. Previous studies discussing blockchain adoption barriers in supply chains have lacked theoretical background. While popular theories such as the technology acceptance model and institutional theory have been used in the literature to explain the underlying motivations of an organisation to adopt a technology, such theories do not explain how managers develop knowledge of the new technology, which eventually shape their actions [32]. There is a paucity of research explaining how organisations perceive the impact of blockchain on their firms and, ultimately, the challenges they face. After carefully analysing the theories in the current literature, we adopted four different theoretical lenses to understand the blockchain phenomena in contemporary financial supply chains and investigate its adoption barriers. The theoretical foundation for this study is the sensemaking theory, force field theory, resource-based view (RBV), and information processing theory (IPT). Figure 5 illustrates the theoretical framework linking the challenges caused by the barriers and the theoretical concepts originating from the four theories.

Most of the existing literature on technology adoption emphasises the implementation phase of the technology adoption process, focusing less on the pre-adoption phase [33]. Adopting a new technology involves significant financial investment and requires changes in the existing business operations. When the disruptive effects of the emerging technology are unpredictable, and its benefits are unclear, sensemaking theory is critical [34]. It helps the firms to make crucial decisions regarding technology adoption. During the pre-adoption stage of blockchain adoption, the SMEs have to make huge investments and bring various organisational changes. Therefore, a sensemaking process can help the firms make appropriate decisions regarding adopting blockchain technology [32]. When adopting radical innovations such as blockchain technology becomes challenging for organisations, managerial sensemaking plays a vital role in inducing the strategic options that organisations can use to shape their future actions [35].

The lack of adoption of blockchain technology can also be explained with the help of force field theory [36]. Force field theory is considered as the theoretical backbone of barrier analysis in supply chain management. Hence, the present study adopts this theory to address the barriers to blockchain adoption in SCF. According to Lewin’s theory, organisational transformation and change incorporate three steps: unfreezing, change, and refreezing. Kouhizadeh et al. [23] argue that firms need to overcome resistant forces for change. These resisting forces or barriers may stem from various internal and external sources.

In pursuit of a higher level of traceability and visibility in supply chain finance transactions, blockchain is a promising technology to drive operational efficiency and competitive advantage. According to Koh et al. [37] resource-based view (RBV) conceptualises the resource efficiency of the firms. The RBV theory explains the skills, capabilities, technology, and processes required to implement blockchain technology in SMEs to boost their competitive advantage. Analysing blockchain through the lens of RBV evaluates the required resources that facilitate the implementation of this technology and helps in understanding the missing capabilities and skills in the organisations [37, 38]. The SMEs’ inadequate resources and the employees’ lack of skills make it challenging for the firm to adopt blockchain technology. RBV provides a theoretical foundation for explaining the dynamic re-adaption of current capabilities of the firms to drive competitive advantage by adopting blockchain in SCF processes.

Information processing theory (IPT) complements the understanding of the effects of blockchain on the SCF processes. The SCF information is used to reduce the firms’ capital and investment risk within the supply chain and improve their financing decisions. The uncertain environment and inadequate management of information processing may jeopardise the integration of the financial supply chains [39]. Information processing theory postulates that firms can alleviate environmental uncertainty by increasing their competencies in gathering, processing, and acting on the information collected from the surroundings. Blockchain provides significant visibility in the supply chain network and ensures data transparency, traceability, and security. This helps the firms improve their information processing capability, eventually enabling them to make sound financial decisions and arrange capital at a lower cost of capital.[40]. The information processing capability of an organisation is affected significantly by the organisational flexibility. The organisational barriers and technology barriers restrict the firm from efficiently collecting and processing information using blockchain technology. Additionally, the security barriers discourage the firms to share commercially sensitive data on blockchain due to fear of losing control over data and other security concerns. All these factors adversely affect the information processing capability of the firms, due to which it loses its competitive advantage in the market.

## Methodology

The study applies a hybrid methodology combining fuzzy-AHP, sensitivity analysis, and fuzzy- DEMATEL. Fuzzy-AHP is used for ranking the barriers and sub-barriers based on their significance, whereas fuzzy DEMATEL highlights the cause and effect relationships among them. In order to evaluate the ranks of particular barriers by making small changes in their weights, the study employs sensitivity analysis. Sensitivity analysis is a crucial tool to ensure the validity of a developed model [41]. The AHP methodology has been extensively used by researchers in different sectors for solving complex multi-criteria decision-making problems [42–45]. However, AHP is incapable of dealing with ambiguity, uncertainty, imprecision, and biases of the decision-makers [44, 46, 47]. To manage these issues and handle the vagueness of the human decision-making practice, the study uses fuzzy set theory with AHP [47]. Moreover, the AHP does not determine the contextual relationships between the barriers, which is studied using fuzzy-DEMATEL [48]. Table 1 shows some studies using the MCDM techniques used in this study that eventually substantiate the rationale of using the fuzzy AHP, fuzzy DEMATEL and sensitivity analysis in this research. The procedural steps followed in fuzzy AHP methodology are explained in Sect. 4.1.

**Table 1:** This table gives an overview of the previous studies that use the methodologies used in this study

Title	Methodology	Application	Reference
Global supplier selection: a fuzzy-AHP approach	F-AHP	This method uses F-AHP to tackle the decision variables involved in the global supplier selection	[47]
Benchmarking health-care supply chain by implementing Industry 4.0: a fuzzy-AHP-DEMATEL approach	F-AHP and F-DEMATEL	The study prioritises and highlights the inter-relationships between the factors affecting the healthcare supply chains	[49]
A fuzzy analytic hierarchy process-based analysis for prioritization of barriers to offshore wind energy	F-AHP and sensitivity analysis	The study identifies and prioritise the barriers to the growth of offshore wind energy in India using F-AHP The study also uses sensitivity analysis to verify the robustness of the developed model	[50]
Blockchain implementation for circular supply chain management: Evaluating critical success factors	AHP and DEMATEL	The study identifies the critical success factors of blockchain implementation in circular supply chain management The study employs AHP and DEMATEL to discover the priorities and relationships among the success factors	[51]
Identification and analysis of circular supply chain management practices for sustainability: a fuzzy-DEMATEL approach	F-DEMATEL	The study identifies the circular practices in circular supply chain management in Indian auto sector F-DEMATEL is used to find the cause and effect relationship between the circular practices	[52]

## Data analysis

In order to collect data, academicians and practitioners knowledgeable in blockchain and supply chains were approached to identify the key barriers, prioritise them and evaluate their cause and effect relationship. A questionnaire-based survey was conducted amongst 58 supply chain professionals to identify the key barriers and sub-barriers to blockchain adoption in supply chain finance. The experts were asked to rank the barriers based on their significance on a five-point rating scale in this survey. The experts were also asked to suggest any new barriers relevant to the study. We shortlisted 22 sub-barriers grouped in 6 categories based on the survey responses. The experts also added three new barriers to the study. Finally, we got 25 sub-barriers categorised under six barrier categories, which are given in Table 2.

**Table 2:** Description of barriers to blockchain adoption in SCF practices by SMEs

Barrier	Sub-barrier	Description	Reference
Technology barriers (TB)	Lack of technological infrastructure in SMEs (TB1)	The general technology infrastructure in India is still at the development stage. Therefore framework to support blockchain in SMEs in India is limited and cost-prohibitive. A good internet connection which is crucial for the successful implementation of blockchain technology is not assured due to poorly developed data infrastructure in many parts of the country	[21, 26]
	Lack of scalability and speed of blockchain system (TB2)	The blockchain technology exhibits significantly lower transaction performance than current systems due to its inadequate scalability. The scalability limits of blockchain are related to the size of the data on the blockchain, the transaction processing rate, and the latency of data transmission which leads to slow transaction speed. E.g. existing blockchain can process 7 transactions per second (tps) in comparison to 500 and 2000 tps processed by Visa and Paypal respectively	[26, 53, 54]
	Lack of Interoperability (between different blockchains, existing technology and legacy systems) (TB3)	Interoperability is the ability to operate and transact on different systems. For successful mass adoption of blockchain, the integration of blockchain platforms with predominant legacy systems and regular IT applications is essential. Moreover, different blockchain systems should be compatible enough to perform transactions among each other. But blockchain ecosystem is siloed and cannot communicate properly with other systems	[55, 56]
	Lack of automation of invoicing and payment processes in SMEs (TB4)	The lack of automated payment processes in SMEs makes it difficult to implement the blockchain-based SCF platform. There are many SMEs in India that uses paper-based invoices, which is a disadvantage while implementing blockchain technology	[55]
	Lack of standardisation (TB5)	Lack of standardisation is one of the most significant shortcomings of blockchain technology. Standards constitute agreed and documented way of conducting business. Standards are required in establishing market confidence to support the rollout of blockchain technology. Standardization can further advance the development of blockchain by providing internationally agreed ways of working, stimulating greater interoperability, speedier acceptance and enhanced innovation	[20, 26]
	Lack of infrastructure providers (TB6)	Blockchain is an emerging technology. Therefore there is a shortage of firms building blockchain infrastructure in India which further impedes the adoption of blockchain in SMEs in India	Added by expert
Organisational barriers (OB)	Resistance to convert to new systems (OB1)	Change is a painful process and is always accompanied by resistance. For the deployment of blockchain technology, some changes are required in traditional business processes. The involved stakeholders may be reluctant to participate in this process. Moreover, the hesitation to reveal information from some partners may limit the full benefits of adopting blockchain technology and hinder the successful implementation of this technology	[18, 55]
	Lack of workforce specialised in Blockchain technology (OB2)	Any emerging technology, in its early years of adoption, requires technically expert workforce to ensure its implementation. The requisite numbers of such specialised workers are in short supply at present in India. Limited technical competence and knowledge of using blockchain technology act as a barrier of adopting this new technology into the supply chain finance	[55, 56]
	Problems in collaboration, communication and coordination in the supply chain (OB3)	Supply chain partners with different operational objectives and priorities may lack collaboration which can disturb the supply chain operations and implementation of blockchain technology in the firm. If the supply chain partners are geographically dispersed, the communication challenges can get worse	[23, 57]
	Lack of information disclosure policy between supply chain partners (OB4)	Supply chain partners are reluctant to share the sensitive financial data on blockchain due to its open and distributed architecture. Additionally, lack of privacy policies related to data usage in supply chains makes it challenging for the supply chain partners to share data with each other. There should be standard rules and policies for information sharing among supply chain partners so that they can collaborate and implement this new technology effectively	[23, 58]
	Lack of collaboration for creating consortium blockchain (OB5)	The consortia creation among the multiple stakeholders in supply chain helps to create, deploy and scale industry-wide solutions. Lack of trust and coordination among the supply chain partners impedes the process of consortia creation. The consortium model allows the supply chain partners to balance the benefits and costs and take advantage of blockchain technology collaboratively. Consortia creation fuels the financial requirement for unlocking the true potential of technology	[59, 60]
External barriers (EB)	Market competition and uncertainty about using blockchain technology (EB1)	Applying blockchain technology may affect the market competitiveness of the firm as it is a complex and time-consuming task. Blockchain users are always worried whether their supply chain partners and payment merchants will adopt the new technology in future. Otherwise, the whole exercise of implementing blockchain can turn futile	[23, 61]
	Legal and regulatory challenges (EB2)	Blockchain users and network operators may experience legal and regulatory uncertainty because there is no settled law of blockchain, so the organisations are translating the existing legal concepts in light of blockchain technology. Legal and regulatory risks include know-your-customer (KYC), anti-money laundering (AML), intellectual property (IP) protection and data privacy and security regulations	[55, 53, 56, 62]
	Lack of qualified blockchain developers (EB3)	There is a dearth of trained blockchain developers across the globe. Although the technical aptitude required for blockchain programmes is very similar to the popular programming languages such as java and python, the number of blockchain developers globally are far less than the java developers	[55, 53, 62]
	Lack of Ecosystem collaboration with blockchain (EB4)	Collaboration is a critical success factor in a blockcahin project because it always involve cross-enterprise workflows. Since information in a blockchain project will usually be shared across multiple supply-chain participants, it is important to consider how the ecosystem will operate and be governed. Whereas, a lack of ecosystem planning can hamper the blockchain projects in supply chain finance	[53, 60]
Knowledge barriers (KB)	Lack of blockchain knowledge (KB1)	Lack of knowledge about handling and managing the blockchain-based SCF projects acts as barrier to its adoption. The existing employees who are trained to work on the centralised systems such as ERP may require training from the area expert to use such platforms	Added by expert
	Lack of understanding of cost, ROI and financial losses (KB2)	SMEs are pondering on the costs, financial losses and return on investment (ROI) in the blockchain-based business models. The incomplete information about the costs and benefits of these projects discourage the SMEs to adopt this technology. Additionally, there may be a financial loss to the blockchain users due to absence of trusted third party or due to incorrect representation of commercial contracts in the smart contract code	[56]
	Blockchain configuration decision (KB3)	One of the most crucial decision for the organisations launching blockchain projects is whether to use a public or private blockchain. This decision will affect the security, functionality and compatibility with other member’s systems. Additionally, selection of the consensus algorithms (e.g. proof of work and proof of stake) is also a big challenge for the organisations	[26]
Security barriers (SB)	Data protection and privacy concerns (SB1)	Blockchain faces some fundamental privacy issues by virtue of its design. Each transaction can be traced in this distributed ledger. The user’s anonymity seems to be compromised as the technology is highly transparent. Therefore none of the supply chain actors will be willing to share commercially sensitive data on blockchain. In some countries data protection and privacy are enforced by legislation e.g. the General data protection regulation in European union. But blockchain may adversely affect the data protection rights	[26, 53]
	Data security concerns (SB2)	Blockchain can be detrimental to a business because of some security issues. The most worrisome threat to security is the possibility of a 51-percent attack, in which one mining entity grabs the control of blockchain and manipulates it. Other areas of concern are DDoS attacks, DNS attack, mempool attack, double spending, consensus delay, etc	[63, 53, 56]
	Data integrity concerns (SB3)	In supply chains data integrity indicates towards the timeliness, completeness and accuracy of the data over its entire lifetime. Data should be preserved from the point of data creation to the point of usage on the blockchain. Blockchain prevents the modification of data once it is entered on the chain. If inaccurate data is entered on the blockchain then there is no benefit of making it immutable. It will be ‘garbage in, garbage out’	[55]
Financial barriers (FB)	Huge initial capital investment for infrastructure and energy resources (FB1)	Blockchain requires huge capital investment in new hardware and software by the organisations and their network partners in the supply chain. Moreover, once implemented, these systems consumes enormous amount of energy, which adds to the total cost of the organisations. Therefore, high initial investment may impede the SME owners to adopt this technology	[55]
	Lack of financial resources (FB2)	While adopting and implementing a new technology, cost is the most critical factor for a business. SMEs may find it difficult to get started with this new technology because of its lack of funds. If it takes a long time to recover the cost of implementing blockchain, SMEs may drop the idea of adopting it. For SMEs, affordability can be a major issue	[23, 58]
	Complex tax implications around digital assets (FB3)	The tax rules on digital assets are very inconsistent on among various jurisdictions. The tax implications around digital assets are very complex. SMEs may need to consult with local tax specialists in the jurisdiction for calculating the tax liability and compliance reporting requirement	[60]
	Audit concerns (FB4)	Blockchain technology presents a whole new set of challenges for auditors. The auditing process of a technology which is designed for privacy is very complex. The transactions on a blockchain platform are irreversible. Therefore, it is important for the auditors to assess that automated controls are effective in validating the transactions. Additional expertise in auditors will be required to satisfy the expectations of the business owners and stakeholders	Added by expert

**Fig. 6:**
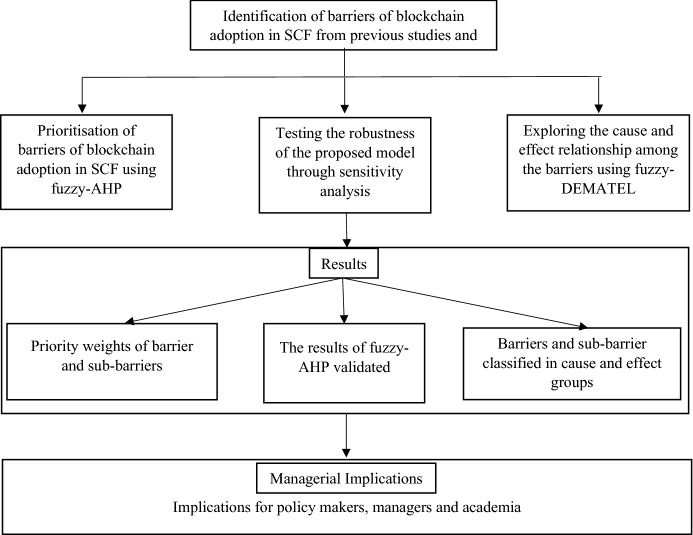
The proposed research framework

In the next step, 30 experts in supply chains and blockchains were contacted to know the priority and the relationships between these barriers and sub-barriers. However, 15 experts agreed to participate as decision makers in this task. The group size of experts can affect the results of data analysis in the study. According to Gumus [64], 5–50 experts are considered optimum for such analysis. The demographic profile of the experts is illustrated in Appendix 1. The methodology adopted in this study is given in Fig. 6.

### Prioritising barriers to blockchain adoption in supply chain finance: Fuzzy AHP

Fuzzy AHP is an advanced analytical method developed from the traditional AHP proposed by Saaty [65]. It is a combination of AHP and the concepts of fuzzy set theory. This method employs the fuzzy rating scales to assess the intensity of the variable in the given attributes [66]. Fuzzy AHP can deal with the imprecision and subjectivity in the judgments given by the decision-makers. Range assessments are considered more secure than fixed assessments by the decision-makers [44]. The computational process of fuzzy AHP followed in this study is explained in the following steps:

*Step* 1 Identifying the key barriers and sub- barriers to the adoption of blockchain in SCF: To identify the key barriers, past studies in this area have been reviewed, and six main barriers and 26 sub-barriers were identified. These barriers were then put for deliberation of practitioners and academicians to add or eliminate any barrier. Finally, the experts shortlisted 22 sub-barriers and added three new barriers, giving us 25 sub-barriers classified into six main categories.

*Step* 2 Employ fuzzy set theory to deal with the imprecision in the experts’ judgments: Fuzzy set theory, proposed by Zadeh [67], helps solve problems of fuzzy phenomena by overcoming the vagueness of human judgments during decision making. The study opts for triangular fuzzy numbers (TFNs), which are considered to be most suitable for evaluating linguistic variables in industrial problems [44]. The fuzzy set theory assigns different degrees of membership to the objects of membership function. The degree of membership is represented by numbers ranging between 0 and 1 known as fuzzy numbers. A triangular fuzzy number Ã with membership function µÃ^(x)^: X → [0, 1] can be represented as follows:

$$\mu \tilde{{\rm A}}\left( {\rm x} \right) = \left\{ {\begin{array}{*{20}c} {\left( {x - l} \right)/\left( {m - l} \right),} & {l \le x \le m} \\ {\left( {u - x} \right)/\left( {u - m} \right),} & {m \le x \le u} \\ {0,} & {otherwise} \\ \end{array} } \right.$$

where l, m, and u are the lower, medium, and upper values of the fuzzy number Ã. The triangular fuzzy number Ã may be represented as (l, m, u).

**Fig. 7:**
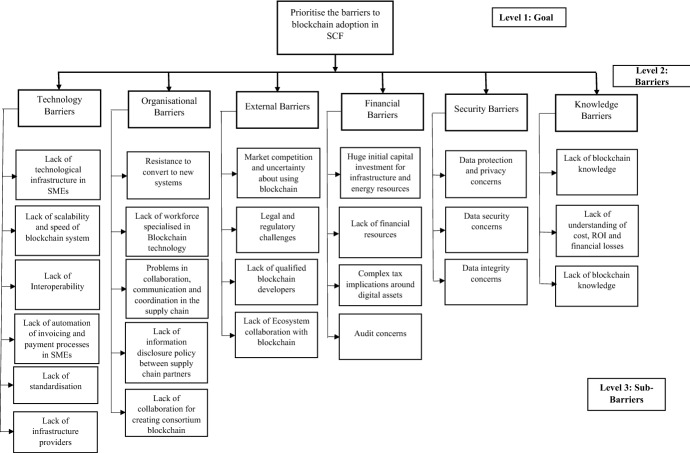
The hierarchy structure of the barriers

*Step* 3 Build a hierarchical structure of the barriers: In this step, a hierarchical structure of the problem is presented, comprising three levels. Level 1 indicates the goal of the given problem; level 2 shows the major barriers to the adoption of blockchain in SCF, and level 3 describes the sub-barriers categorised within each main barrier. Figure 7 presents the hierarchical structure of the barriers.

*Step* 4 Compute the pairwise comparison matrices for the barriers and sub-barriers: Fuzzy pairwise comparison matrices are prepared based on the judgement of the experts through the fuzzy linguistic scale provided in Table 3. The experts rated their preferences on one barrier over the other in the fuzzy-AHP questionnaire. The final fuzzy pairwise comparison matrix, constructed after converting the linguistic responses of the experts in to triangular fuzzy numbers can be illustrated as K = [a_ij_]_mxn_ where the fuzzy entries in the matrices are represented by a_ij_ = (p_ij_, q_ij_, r_ij_). These fuzzy numbers fulfils the following property:

$$a_{ij} = \frac{1}{{a_{ji} }},b_{ij} = \frac{1}{{b_{ji} }},c_{ij} = \frac{1}{{c_{ji} }}$$

where i and j = 1,2,3,…n.

**Table 3:** The fuzzy linguistic scale for prioritisation of barriers

Linguistic variables	Triangular fuzzy numbers	Inverse triangular fuzzy numbers
Equally important	(1,1,1)	(1,1,1)
Moderately important	(1,2,3)	(1/3,1/2, 1)
Strongly important	(2,3,4)	(1/4, 1/3, 1/2)
Very strongly important	(3,4,5)	(1/5, 1/4, 1/3)
Extremely important	(4,5,6)	(1/6, 1/5, 1/4)

*Step* 5 Calculate the significance weights of the barriers: In this step, the triangular fuzzy numbers of the fuzzy pair-wise comparison matrices are processed to calculate the weights of the barriers’ significance to establish their priority. The calculation requires specific algebraic operations. Therefore, Chang’s extent analysis method is used to calculate the significance weights of the barriers and sub-barriers [46, 47, 68, 69]. Further, some necessary steps used in the calculation are given as follows:

$${\text{S}}_{1} = \left( {4.95, 6.22, 7.73} \right) \times \left( {\frac{1}{37.29},\frac{1}{29.51},\frac{1}{23.71}} \right)$$

$${\text{S}}_{1} = \left( {0.13,0.21,0.33} \right)$$

$${\text{S}}_{2} = \left( {4.17, 5.22, 6.51} \right) \times \left( {\frac{1}{37.29},\frac{1}{29.51},\frac{1}{23.71}} \right)$$

$${\text{S}}_{2} = \left( {0.11,0.18,0.27} \right)$$

$${\text{S}}_{3} = \left( {3.49, 4.31, 5.42} \right) \times \left( {\frac{1}{37.29},\frac{1}{29.51},\frac{1}{23.71}} \right)$$

$${\text{S}}_{3} = \left( {0.09,0.15,0.23} \right)$$

$${\text{S}}_{4} = \left( {3.83, 4.78, 6.22} \right) \times \left( {\frac{1}{37.29},\frac{1}{29.51},\frac{1}{23.71}} \right)$$

$${\text{S}}_{4} = \left( {0.10,0.16,0.26} \right)$$

$${\text{S}}_{5} = \left( {4.20,5.08,6.22} \right) \times \left( {\frac{1}{37.29},\frac{1}{29.51},\frac{1}{23.71}} \right)$$

$${\text{S}}_{5} = \left( {0.11,0.17,0.26} \right)$$

$${\text{S}}_{6} = \left( {3.06,3.89,5.19} \right) \times \left( {\frac{1}{37.29},\frac{1}{29.51},\frac{1}{23.71}} \right)$$

$${\text{S}}_{6} = \left( {0.08,0.13,0.22} \right)$$

$$z^{\prime } \left( {{\text{C}}_{1} } \right) = \min V\left( {{\text{S}}_{1} \ge {\text{S}}_{2} , {\text{S}}_{3} , {\text{S}}_{4} , {\text{S}}_{5} , {\text{S}}_{6} } \right) = \min \left( {1, 1, 1,1,1} \right) = 1$$

$$z^{\prime } \left( {{\text{C}}_{2} } \right) = 0.808$$

$$z^{\prime } \left( {{\text{C}}_{3} } \right) = 0.597$$

$$z^{\prime } \left( {{\text{C}}_{4} } \right) = 0.726$$

$$z^{\prime } \left( {{\text{C}}_{5} } \right) = 0.770$$

$$z^{\prime } \left( {{\text{C}}_{6} } \right) = 0.521$$

In order to establish the weight vectors for main barriers, the obtained values are normalised. The resultant significance weights of the barriers are shown in Table 4. The order of significance of the main barriers is TB—OB—SB—KB—EB—FB.

**Table 4:** Ranking of main barriers to blockchain adoption in scf practices by SMEs

Barriers	Significance weights	Ranking
Technology barrier (TB)	0.2261	1
Organisation barrier (OB)	0.1826	2
Security barrier (SB)	0.1742	3
Knowledge barrier (KB)	0.1643	4
External barriers (EB)	0.1350	5
Financial barrier (FB)	0.1178	6

**Fig. 8:**
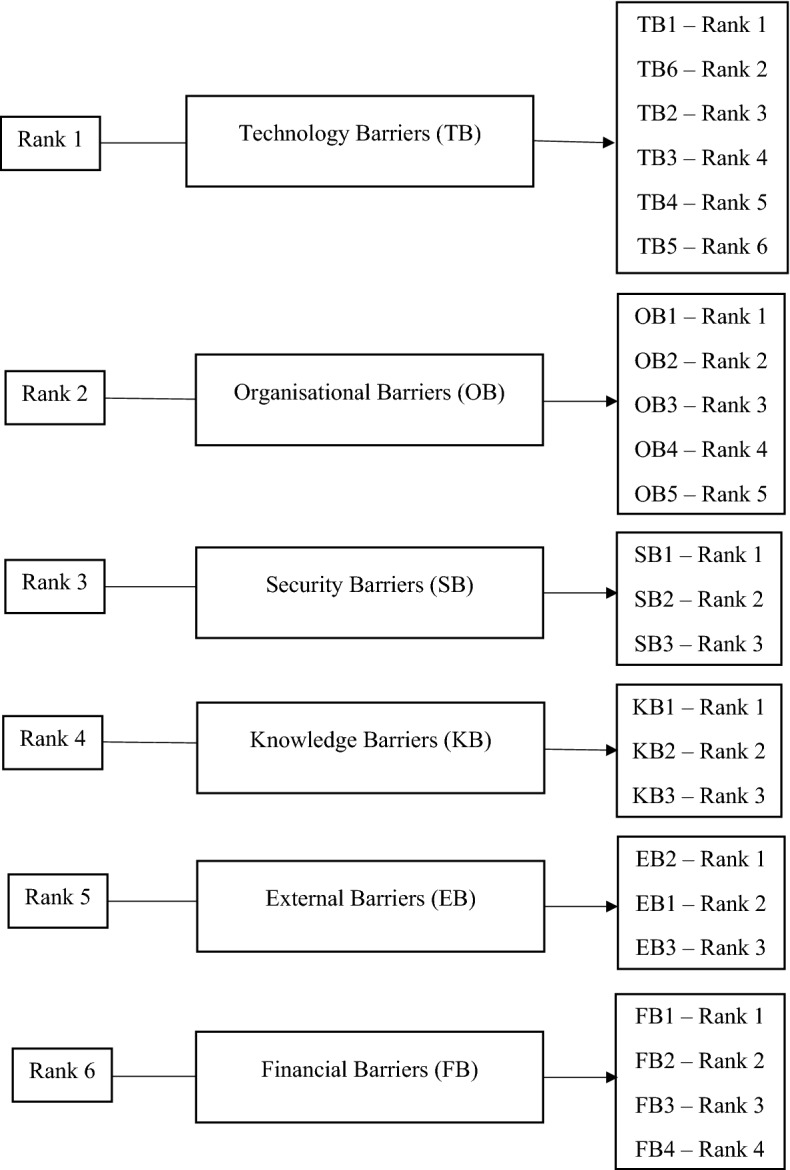
Ranking of barriers and sub-barriers to blockchain adoption in SCF practices by SMEs

The sub-barriers were ranked based on their relative weights and global weights in the next level. The global weights of the sub-barriers were obtained by multiplying the relative weights of each sub-barrier with the preference weights of the barriers. The global ranking of the barriers to blockchain adoption in supply chain finance is summarised in Table 5. Additionally, Fig. 8 illustrates the ranking of the barriers and sub-barriers.

**Table 5:** Relative and global ranking of sub-barriers to blockchain adoption in SCF practices by SMEs

Main barriers	Sub-barriers	Relative weights	Relative ranking	Global weights	Global ranking
Technology barriers (TB)	TB1	0.3405	1	0.0770	3
	TB2	0.1533	3	0.0347	13
	TB3	0.0864	4	0.0195	20
	TB4	0.0833	5	0.0188	21
	TB5	0.0022	6	0.0005	25
	TB6	0.3293	2	0.0745	4
Organisation barriers (OB)	OB1	0.2454	1	0.0448	8
	OB2	0.2259	2	0.0412	10
	OB3	0.2071	3	0.0378	12
	OB4	0.181	4	0.0331	15
	OB5	0.1506	5	0.0275	17
Security barriers (SB)	SB1	0.6263	1	0.1091	1
	SB2	0.3104	2	0.0541	6
	SB3	0.0634	3	0.0110	23
Knowledge barriers (KB)	KB1	0.5544	1	0.0911	2
	KB2	0.3097	2	0.0509	7
	KB3	0.1359	3	0.0223	18
External barriers (EB)	EB1	0.2824	2	0.0381	11
	EB2	0.3268	1	0.0441	9
	EB3	0.2287	3	0.0309	16
	EB4	0.1622	4	0.0219	19
Financial barriers (FB)	FB1	0.5139	1	0.0605	5
	FB2	0.2872	2	0.0338	14
	FB3	0.1313	3	0.0155	22
	FB4	0.0676	4	0.0080	24

### Sensitivity analysis

**Fig. 9:**
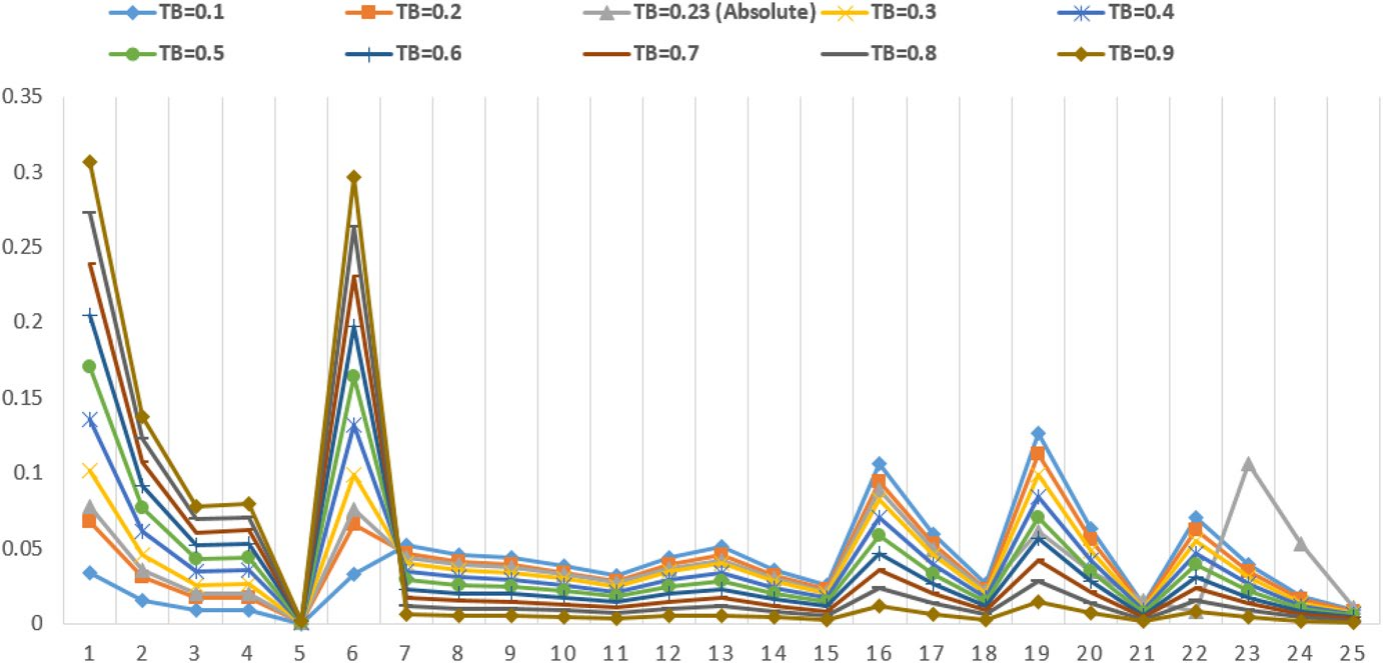
Results of sensitivity analysis for sub-barriers to blockchain adoption in SCF practices by SMEs

Sensitivity analysis is an essential tool to check the validity and robustness of the proposed framework [66, 69]. It helps to analyse the behaviour of a specific model under different types of working environments [44]. In this study, technology barriers (TB) receive the highest priority weight among the other barrier categories. Therefore, it can affect the other barriers. Hence, the weight of the technology barriers was varied from 0.1 to 0.9 to check its impact on the other barriers (Table 6). The results show that the maximum change was observed in the “Organisational barrier (OB)”. The variations in the weight of technology barriers also influence the ranking of the sub barriers, as shown in Table 6. In addition, variation in results is also sketched as given in Fig. 9. In sensitivity analysis, when the weight of TB is 0.1 and 0.2, FB1 and KB1 acquire the first and second rank, respectively, whereas TB5 holds the last rank. It is visible from the results that TB1 and TB6 stand at first and second rank during all the seven trials when the value of TB varies between 0.3 and 0.9. However, the rank of TB5 remains unchanged during the first eight runs of sensitivity analysis and stays at the last rank. The sensitivity analysis results confirm that the technology barriers category is the most important and influences the adoption of blockchain in SCF the most. Therefore, it needs a greater concentration of the management.

**Table 6:** Global priority weights for sub-barriers due to sensitivity analysis

Barriers sub-criteria	TB = 0.1	TB = 0.2	TB = 0.23 (absolute)	TB = 0.3	TB = 0.4	TB = 0.5	TB = 0.6	TB = 0.7	TB = 0.8	TB = 0.9	Barriers sub-criteria
TB1	0.0340	0.0681	0.0783	0.1021	0.1362	0.1702	0.2043	0.2383	0.2724	0.3064	TB1
TB2	0.0153	0.0307	0.0353	0.0460	0.0613	0.0767	0.0920	0.1073	0.1227	0.1380	TB2
TB3	0.0086	0.0173	0.0199	0.0259	0.0346	0.0432	0.0519	0.0605	0.0692	0.0778	TB3
TB4	0.0088	0.0177	0.0203	0.0265	0.0353	0.0441	0.0530	0.0618	0.0706	0.0795	TB4
TB5	0.0002	0.0004	0.0005	0.0007	0.0009	0.0011	0.0013	0.0016	0.0018	0.0020	TB5
TB6	0.0329	0.0659	0.0757	0.0988	0.1317	0.1646	0.1976	0.2305	0.2634	0.2963	TB6
OB1	0.0521	0.0463	0.0442	0.0405	0.0347	0.0290	0.0232	0.0174	0.0116	0.0058	OB1
OB2	0.0459	0.0408	0.0389	0.0357	0.0306	0.0255	0.0204	0.0153	0.0102	0.0051	OB2
OB3	0.0440	0.0391	0.0373	0.0342	0.0293	0.0244	0.0196	0.0147	0.0098	0.0049	OB3
OB4	0.0384	0.0342	0.0326	0.0299	0.0256	0.0214	0.0171	0.0128	0.0085	0.0043	OB4
OB5	0.0320	0.0284	0.0271	0.0249	0.0213	0.0178	0.0142	0.0107	0.0071	0.0036	OB5
EB1	0.0443	0.0394	0.0367	0.0345	0.0295	0.0246	0.0197	0.0148	0.0098	0.0049	EB1
EB2	0.0513	0.0456	0.0425	0.0399	0.0342	0.0285	0.0228	0.0171	0.0114	0.0057	EB2
EB3	0.0359	0.0319	0.0297	0.0279	0.0239	0.0199	0.0160	0.0120	0.0080	0.0040	EB3
EB4	0.0255	0.0226	0.0211	0.0198	0.0170	0.0141	0.0113	0.0085	0.0057	0.0028	EB4
KB1	0.1059	0.0941	0.0887	0.0824	0.0706	0.0588	0.0471	0.0353	0.0235	0.0118	KB1
KB2	0.0592	0.0526	0.0496	0.0460	0.0394	0.0329	0.0263	0.0197	0.0131	0.0066	KB2
KB3	0.0260	0.0231	0.0218	0.0202	0.0173	0.0144	0.0115	0.0087	0.0058	0.0029	KB3
FB1	0.1269	0.1128	0.0617	0.0987	0.0846	0.0705	0.0564	0.0423	0.0282	0.0141	FB1
FB2	0.0629	0.0559	0.0345	0.0489	0.0419	0.0349	0.0279	0.0210	0.0140	0.0070	FB2
FB3	0.0128	0.0114	0.0158	0.0100	0.0086	0.0071	0.0057	0.0043	0.0029	0.0014	FB3
FB4	0.0704	0.0626	0.0081	0.0548	0.0469	0.0391	0.0313	0.0235	0.0156	0.0078	FB4
SB1	0.0394	0.0350	0.1065	0.0306	0.0262	0.0219	0.0175	0.0131	0.0087	0.0044	SB1
SB2	0.0180	0.0160	0.0528	0.0140	0.0120	0.0100	0.0080	0.0060	0.0040	0.0020	SB2
SB3	0.0093	0.0082	0.0108	0.0072	0.0062	0.0051	0.0041	0.0031	0.0021	0.0010	SB3

### Classifying the barriers to blockchain adoption in supply chain finance into cause and effect groups: Fuzzy-DEMATEL

The DEMATEL technique helps analyse complex cause and effect relationships among factors in multi-criteria decision-making problems [45, 70]. It is a decision-making tool based on graph theory proposed at Geneva research Centre [71]. DEMATEL also calculates the strength of relationship among the factors [72] and categorises the factors into cause and effect groups [73, 74]. However, DEMATEL is not capable enough to deal with the problems of uncertainty and vagueness of data and biases of human judgment; therefore, the study uses fuzzy-DEMATEL [45, 70, 72]. Following are the steps followed in fuzzy-DEMATEL analysis:

*Step* 1 Collect inputs from the experts on the effect of one barrier on others: The responses from the experts are collected through a fuzzy-DEMATEL questionnaire. The experts made pairwise comparisons among the barriers, recorded their responses using the fuzzy linguistic scale (Table 7), and formed pairwise comparison matrices.

**Table 7:** The fuzzy linguistic scale for measuring the influence of one barrier on other

Linguistic variable	Preference score	Corresponding triangular fuzzy numbers
No influence (NI)	0	(0, 0, 0.25)
Low influence (LI)	1	(0, 0.25, 0.50)
Medium influence (MI)	2	(0.25, 0.50, 0.75)
High influence (HI)	3	(0.50, 0.75, 1.0)
Very high influence (VHI)	4	(0.75, 1.0, 1.0)

*Step* 2 Develop a direct relationship matrix: Using fuzzy linguistic scales, the pairwise comparison matrices were converted into initial direct relationship matrices. For each expert, a different direct relationship matrix was drawn. These matrices were then converted into fuzzy average direct relationship matrix (A) using Eq. (1)

1 $$A = (a_{ij} ) = \frac{1}{n}\mathop \sum \limits_{k = 1}^{n} a_{ij}$$

here *n* signifies the number of experts. The triangular fuzzy numbers are de-fuzzified into crisp numbers using Eq. (2), which results into fuzzy direct relationship matrix presented in Table 8.

2 $$D_{T} = \frac{1}{6}\left( {l + 4m + u} \right)$$

**Table 8:** Direct relationship matrix of main barrier categories

	TB	OB	EB	KB	SB	FB
TB	0.04	0.5	1	0.75	0.75	0.75
OB	0.5	0.04	0.75	0.75	1	4
EB	0.5	0.75	0.04	0.5	0.5	1
KB	0.75	0.75	4	0.04	0.5	0.5
SB	0.75	0.75	0.75	0.75	0.04	0.75
FB	4	4	0.75	0.5	0.75	0.04

here *l*, *m* and *u* are triangular fuzzy numbers.

*Step* 3 Develop a normalised initial direct relationship matrix: The initial direct relationship matrix was normalised through equations Eqs. (3) and (4).

The normalised direct relationship matrix

3 $$\left( {\text{N}} \right) = {\text{k}}.{\text{A}}$$

4 $$k = \frac{1}{{\max_{1 \le i \le n} \mathop \sum \nolimits_{j = 1}^{n} a_{ij} }}$$

*Step* 4 Develop the total relationship matrix: After computing the normalised direct-relation matrix, the total relationship matrix (T) is calculated using Eq. 5.

5 $$T = N\left( {I - N} \right)^{ - 1}$$

here *I* represents the identity matrix.

**Fig. 10:**
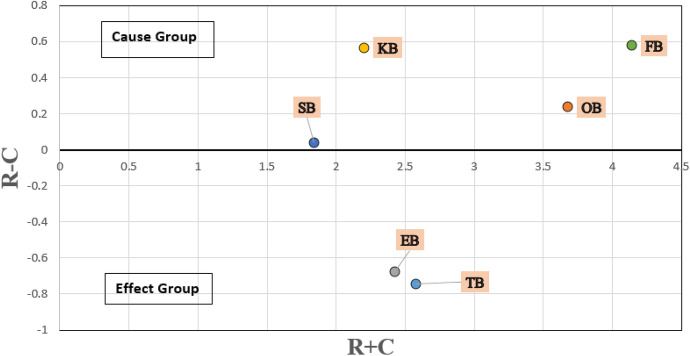
Cause and effect relationship between the barriers to blockchain adoption in SCF practices by SMEs

**Fig. 11:**
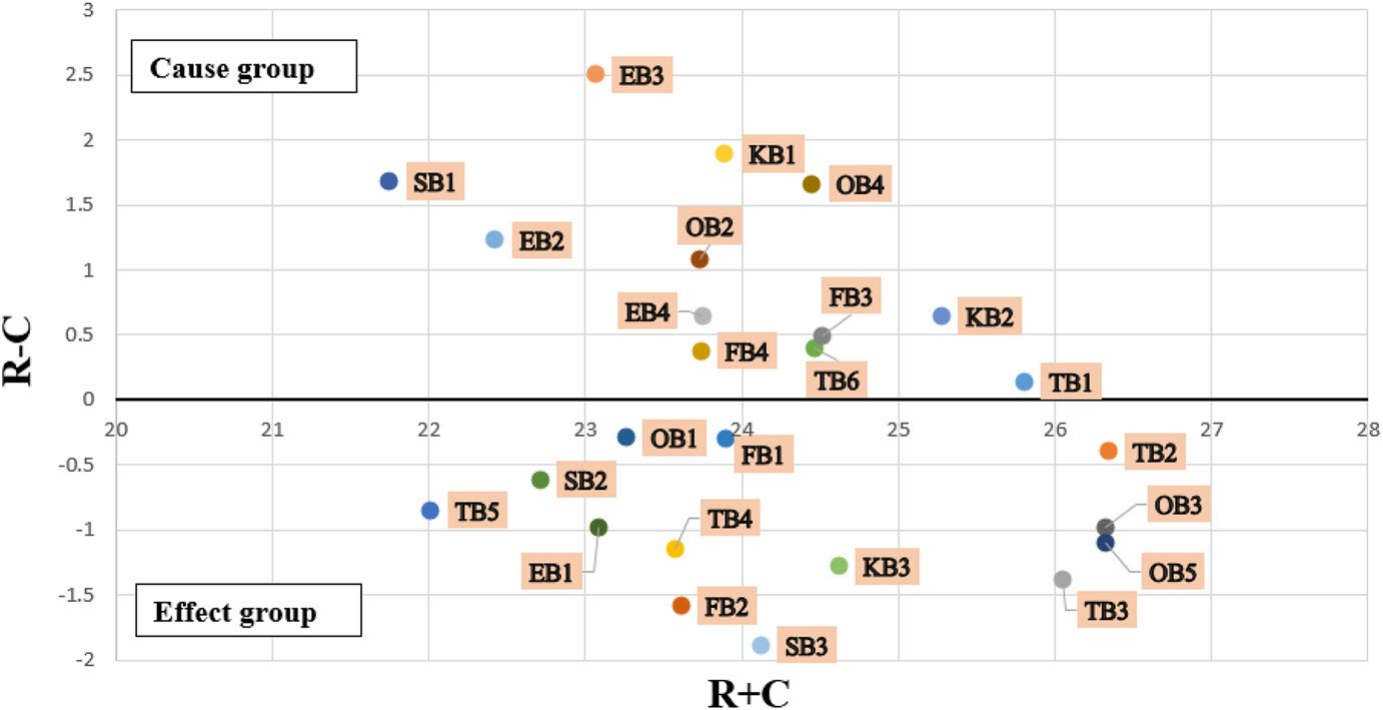
The cause and effect relationship between sub-barriers to blockchain adoption in SCF practices by SMEs

In the total relationship matrix, the sum of all rows (represented by R) and all columns (represented by C) has been calculated using Eq. 6 and Eq. 7, respectively. The values for (R + C) and (R – C) were calculated to classify the barriers into cause and effect categories, as presented in Table 9. (R + C) values reveal the relative importance of one barrier over the other barriers. On the other hand, (R – C) values allow dividing the barriers into cause and effect groups. When the value of (R – C) is positive, the barrier belongs to the cause group, whereas, if the value is negative, the barrier belongs to the effect group [75]. The cause and effect relationship between the barriers and sub-barriers is displayed in Fig. 10 and 11, respectively.

6 $$r_{i} = \mathop \sum \limits_{1 \le j \le n} t_{ij}$$

7 $$c_{i} = \mathop \sum \limits_{1 \le i \le n} t_{ij}$$

**Table 9:** The cause and effect analysis among barriers and sub-barriers to blockchain adoption in SCF practices by SMEs

Barrier	R + C	R – C	Barrier’s attribute	Sub-barrier	R + C	R – C	Sub-barrier’s attribute
TB	2.57	− 0.74	Effect	TB1	25.81	0.14	Cause
				TB2	26.34	− 0.39	Effect
				TB3	26.05	− 1.38	Effect
				TB4	23.57	− 1.14	Effect
				TB5	22.01	− 0.85	Effect
				TB6	24.47	0.39	Cause
OB	3.68	0.24	Cause	OB1	23.26	− 0.29	Effect
				OB2	23.73	1.09	Cause
				OB3	26.32	− 0.98	Effect
				OB4	24.45	1.67	Cause
				OB5	26.32	− 1.10	Effect
EB	2.42	− 0.68	Effect	EB1	23.09	− 0.99	Effect
				EB2	22.42	1.23	Cause
				EB3	23.06	2.50	Cause
				EB4	23.75	0.65	Cause
KB	2.20	0.57	Cause	KB1	23.89	1.89	Cause
				KB2	25.28	0.65	Cause
				KB3	24.62	− 1.28	Effect
SB	1.84	0.04	Cause	SB1	23.89	− 0.29	Effect
				SB2	23.61	− 1.58	Effect
				SB3	24.51	0.49	Cause
FB	4.14	0.58	Cause	FB1	23.74	0.38	Cause
				FB2	21.75	1.68	Cause
				FB3	22.71	− 0.61	Effect
				FB4	24.12	− 1.89	Effect

## Results and discussion

The results of fuzzy-AHP reveal that technology barriers (TB) obtain the first rank, and therefore, occupy the highest priority among all the barriers. This category includes the barriers stemming from the limitations of blockchain technology. It incorporates the technical capability, difficulty, complexity, and availability of blockchain technology [76]. There are six sub-barriers in this category. ‘Lack of technological infrastructure in SMEs’ (TB1) obtains the highest priority. The technology framework supporting blockchain technology is inadequate and cost-prohibitive for SMEs in India [21]. ‘Lack of infrastructure providers’ (TB6) comes second in this category followed by ‘lack of scalability and speed of blockchain system’ (TB2), ‘lack of interoperability between different blockchains, existing technology and legacy systems (TB3), ‘lack of automation of invoicing and payment processes in SMEs (TB4), and ‘lack of standardisation (TB5).

Organisational barriers come second in the priority list and play a critical role in the adoption of blockchain technology. This category identifies the barriers stemming from the internal activities of organisations and supply chain partners’ relationships. On an inter-organisation level, its challenging to manage the relationships between supply chain partners when adopting innovative technologies [20]. This category has five specific barriers. ‘Resistance to convert to new systems ‘(OB1) occupies the highest priority. When organisations transform to new systems, it may change organisational culture that leads to resistance from the employees [77]. ‘Lack of workforce specialised in Blockchain technology’ (OB2) holds the second rank in the priority list followed by ‘problems in collaboration, communication and coordination in the supply chain (OB3)’, lack of information disclosure policy between supply chain partners (OB4) and ‘lack of collaboration for creating consortium blockchain (OB5).

Security barriers (SB) occupy third place in the priority list. In terms of security and scalability, blockchain is still considered an immature technology [63]. Therefore it faces numerous security barriers. ‘Data protection and privacy concerns’ (SB1) hold the highest priority. ‘Data security concerns’ (SB2) comes next to SB1. Finally, ‘Data integrity concerns’ (SB3) comes last in the list. Knowledge barriers acquire the fourth importance level. Limited knowledge and technical expertise of using blockchain technology act as a barrier in adopting this technology into supply chain finance. Both technical and non-technical employees must be knowledgeable in implementing blockchain projects [78]. There are three specific barriers in this category. 'Lack of blockchain knowledge’ (KB1) got the highest priority among them. Based on the priority rank ‘lack of understanding of cost, ROI, and financial losses’ (KB2) comes second and ‘blockchain configuration decision’ (KB3) comes last in the priority list.

External barriers occupy fifth rank. This barrier category presents the challenges stemming from external stakeholders, institutions, industries, and governments. In this category, ‘Legal and regulatory challenges’ (EB2) hold the highest priority. ‘Market competition and uncertainty about using blockchain’ (EB1) are ranked next to EB2 followed by ‘lack of qualified blockchain developers’ (EB3). Financial barriers hold the last place on the priority list and play a crucial role in adopting blockchain technology. It suggests that implementing blockchain technology is very costly for SMEs, and a lack of funds impedes its adoption. There are four specific barriers in this category. Among them, ‘Huge initial capital investment for infrastructure and energy resources’ (FB1) got the highest priority. ‘Lack of financial resources’ (FB2) comes next in this category followed by ‘Complex tax implications around digital assets’ (FB3) and ‘Audit concerns’ (FB4).

DEMATEL traces the cause and effect relationships between the barriers. Table 9 shows the classification of barriers and sub-barriers into cause and effect groups based on R – C values. The barriers in the cause group are usually independent and drive the effect group barriers [21]. According to the fuzzy-DEMATEL analysis four barriers (‘organisational barriers’, ‘knowledge barriers’, ‘security barriers’, and ‘financial barriers’) belongs to cause group, and must be worked upon for accelerating blockchain adoption in SCF by SMEs. The cause group barriers are the independent barriers having direct effect on the system. The effect group barriers comprise of ‘technology barriers’ and ‘external barriers’. R + C values reveal the importance of each barrier. Financial barriers receive the highest R + C value, which indicates that it is a highly influential barrier. The adoption of this technology requires huge investment which is expensive for organisations and their supply chain partners [20].

## Implications for managers and policy makers

Blockchain has the potential to transform the functioning of SMEs. The technology can overcome SMEs’ long-standing constraints in accessing credit. However, blockchain is in the nascent stage; its adoption rate in SMEs is meagre. The study results reveal that technology and organisational barriers are the most significant barriers of blockchain adoption in SMEs’ SCF practices. Moreover, the findings of the study are in line with the two government reports on blockchain by NITI ayog (the policy think tank of the government of India) and by the ministry of electronics and information technology. Additionally, the survey conducted on blockchain by Deloitte in the year 2020 also confirms the study’s findings. Blockchain is one of the top five strategic priorities for the majority of the survey respondents. Globally, there was a substantial jump in blockchain production in 2020 [55]. The findings of this study have noteworthy implications for managers and policymakers:

### Implications for managers

#### Creating awareness of barriers

This paper presents the key barriers to blockchain adoption by Indian SMEs in SCF. Managers can use the knowledge of key barriers in developing a strategy to overcome the barriers and eventually adopt blockchain in SCF practices.

#### Focus on the high priority barriers

The fuzzy-AHP prioritises the barriers that help the managers to target the most critical barriers. Technology barriers got the highest weightage implying that SMEs’ managers should focus the most on improving the technology infrastructure of their firms.

#### Target the cause group barriers

The DEMATEL-based tagging of the barriers into cause and effect groups helps the managers to control the cause group barriers to mitigate their effect for the successful implementation of blockchain technology.

### Implications for policymakers

#### Development of nation-wide blockchain infrastructure

In India, a national-level blockchain framework can facilitate in scaling the blockchain applications and introduce shared infrastructure for the organizations [53]. Therefore, policymakers should focus on developing infrastructure spread across multiple zones in the country, helping in hosting blockchain platforms. Such an indigenous blockchain platform can reduce the cost of blockchain adoption for SMEs and accelerate its adoption.

#### Promoting research and development

The government needs to promote research and development in blockchain, along with skilling workforce and students [79]. Funding support should be extended by the government to facilitate premier R & D and academic institutions in the country to initiate research activities in the core research areas of blockchain, like interoperability, scalability, data security, and privacy.

#### Building confidence in SMEs

SMEs need to be convinced of the advantages of blockchain technology, and the resistance to adopt this technology should be eliminated. SMEs and their supply chain partners are sceptical about the use of blockchain platforms due to the issues related to privacy, security, interoperability, and automation of invoices. Therefore, the blockchain developer community should resolve these issues to facilitate the user experience which will eventually fuel the adoption rate of blockchain in SMEs.

## Conclusion and limitations

Blockchain technology has the potential to help SMEs overcome long-standing challenges by reducing transaction costs and information asymmetry and facilitating trade and access to credit. SMEs in India face numerous barriers to adopting blockchain technology in SCF practices. Therefore, the study investigates the prominent barriers to adopting blockchain technology in SCF in Indian SMEs. Through extensive literature search and the opinion of the domain experts from academia and industry, 25 sub-barriers were identified under six main barriers. As a methodological contribution, the study employs an integrated approach of Fuzzy-AHP, Sensitivity analysis, and Fuzzy DEMATEL. Fuzzy AHP illustrates the significance of the barriers; sensitivity analysis validates the developed model, and fuzzy-DEMATEL examines the cause and effect relationship between the identified barriers.

Findings of this research reveal that technology barriers have the highest priority, followed by organisational barriers and security barriers. This brings light to the fact that SCF and blockchain practitioners should work on these barriers on a priority basis so that the adoption of blockchain technology can be accelerated to enhance the efficiency of SMEs. Moreover, SMEs should reshape their organisational culture to facilitate the blockchain adoption. Additionally, by implementing the fuzzy-DEMATEL methodology, four barriers have been categorised into cause groups: organisational barriers, knowledge barriers, security barriers, and financial barriers. At the same time, technology barriers and external barriers have been identified as the effect group barriers. The categorisation of these barriers would aid the managers of SMEs to control the barriers in the cause group and reshaping them to implement blockchain technology in SCF practices successfully. In the end, the developed model is tested for its robustness by implementing sensitivity analysis.

The novelty of the research is twofold; first and foremost, this is the first study investigating the blockchain adoption barriers in SCF practices in the Indian context. The second aspect lies in the integrated research methodology used in the study. The three methods complement each other and comprehensively analyse the identified barriers.

The present study bears some limitations, which open the prospects for future research. First, the study has been conducted concerning SMEs in India. The research implications may vary to a certain extent in the context of SMEs in developed nations. Therefore, future researchers may investigate the barriers faced by SMEs of developed nations and compare the results with this study. Second, it is a qualitative study based on experts’ opinions. The researchers may undertake empirical research and validate this research in the future. Third, although the study identifies the most comprehensive set of barriers, in the future, additional barriers may appear, and some of the existing barriers may also become obsolete with rapidly changing technology. Therefore, future researchers have an opportunity to identify new barriers that affect the adoption of blockchain by Indian SMEs in SCF.

## Appendix 1

Demographic profile of the respondents.

**Table:**

Expert designation	Education	Experience (in years)	Service sector
Regional head (Eastern Hemisphere)	M.Sc(Hon) & MBA	38	Oil & gas
Senior manager	M. Tech	16	Food supply chain
Warehousing planning manager	M. Tech	8	Warehousing
Manager	PhD	15	Manufacturing
Project manager	M. Tech	4	Oil & gas
Maintenance engineer	BE MECH	6	Pharmaceutical
Associate professor	DSc	23	Academics
Assistant professor	PhD	5.5	Academics
Senior manager	MBA	11	Consulting
Engineer	B.Tech	8	Manufacturing
Junior manager	B.Tech	8	Manufacturing
Senior manager	M.Sc	10	Supply chain management
Assistant professor	PhD	16	Supply chain management
Assistant professor	PhD	8	Education
Senior manager	M.Tech	10	Manufacturing
